# Drinking-Water Nitrate, Methemoglobinemia, and Global Burden of Disease: A Discussion

**DOI:** 10.1289/ehp.7216

**Published:** 2004-07-22

**Authors:** Lorna Fewtrell

**Affiliations:** Centre for Research into Environment and Health, Crewe, Cheshire, United Kingdom

**Keywords:** burden of disease, drinking water, methemoglobinemia, nitrates

## Abstract

On behalf of the World Health Organization (WHO), I have undertaken a series of literature-based investigations examining the global burden of disease related to a number of environmental risk factors associated with drinking water. In this article I outline the investigation of drinking-water nitrate concentration and methemoglobinemia. The exposure assessment was based on levels of nitrate in drinking water greater than the WHO guideline value of 50 mg/L. No exposure–response relationship, however, could be identified that related drinking-water nitrate level to methemoglobinemia. Indeed, although it has previously been accepted that consumption of drinking water high in nitrates causes methemoglobinemia in infants, it appears now that nitrate may be one of a number of co-factors that play a sometimes complex role in causing the disease. I conclude that, given the apparently low incidence of possible water-related methemoglobinemia, the complex nature of the role of nitrates, and that of individual behavior, it is currently inappropriate to attempt to link illness rates with drinking-water nitrate levels.

The Global Burden of Disease project, coordinated by the World Health Organization (WHO), is an attempt to quantify and compare the level of illness at both world and regional levels. This can be done on a disease-by-disease basis (Murray and Lopez 1996) or in relation to various risk factors such as malnutrition; exposure to poor water, sanitation, and hygiene; or indoor air pollution (Murray and Lopez 1996; Prüss et al. 2002; WHO 2002). Information relating to environmental risk factors, such as the amount of illness attributable to lead in the environment (Fewtrell et al. 2004), can be very powerful in terms of informing policy decisions. Disease burden, in relation to environmental risk factors, is generally determined by establishing the exposure of the population (on a regional basis) to the chosen risk factor and combining these data with exposure–response relationships for the selected health outcomes to estimate the number of people affected with each outcome. This may then be converted into disability-adjusted life years, accounting for the severity and duration of each health outcome.

Nitrate pollution of drinking water (which has been linked with certain health outcomes) is known to be increasing (Croll and Hayes 1988; Tandia et al. 2000; WHO 1985; Young and Morgan-Jones 1980). WHO therefore considered it useful to determine whether it was possible to establish a disease burden estimate.

## Health Outcomes

Nitrate is a naturally occurring ion, which makes up part of the nitrogen cycle. The nitrate ion (NO_3_^−^) is the stable form of combined nitrogen for oxygenated systems. Although it is chemically unreactive, it can be microbially reduced to the reactive nitrite ion. Nitrate has been implicated in methemoglobinemia and also a number of currently inconclusive health outcomes. These include proposed effects such as cancer (via the bacterial production of *N*-nitroso compounds), hypertension, increased infant mortality, central nervous system birth defects, diabetes, spontaneous abortions, respiratory tract infections, and changes to the immune system [Centers for Disease Control and Prevention (CDC) 1996; Dorsch et al. 1984; Gupta et al. 2000; Hill 1999; Kostraba et al. 1992; Kozliuk et al. 1989; Malberg et al. 1978; Morton 1971; Super et al. 1981]. Although the role of *N*-nitroso compounds and nitrite in the promotion of cancer would appear to be incontrovertible, the evidence relating to the role of nitrates is less clear (Pobel et al. 1995). Thus, methemoglobinemia was the only health outcome I considered further in this investigation.

Methemoglobin (MetHb) is formed when nitrite (for our purposes, formed from the endogenous bacterial conversion of nitrate from drinking water) oxidizes the ferrous iron in hemoglobin (Hb) to the ferric form (Fan et al. 1987). MetHb cannot bind oxygen, and the condition of methemoglobinemia is characterized by cyanosis, stupor, and cerebral anoxia (Fan et al. 1987). Under normal conditions, < 2% of the total Hb circulates as MetHb (Fan et al. 1987). Signs of methemoglobinemia appear at 10% MetHb or more, as shown in Table 1 [Craun et al. 1981; Kross et al. 1992; National Academy of Sciences (NAS) 1981]. Symptoms include an unusual bluish gray or brownish gray skin color, irritability, and excessive crying in children with moderate MetHb levels and drowsiness and lethargy at higher levels (Brunning-Fann and Kaneene 1993). Diagnosis is through the observation of chocolate-colored blood or a laboratory test showing the presence of elevated MetHb levels (Brunning-Fann and Kaneene 1993).

Infant methemoglobinemia was first linked to nitrates in drinking water by Hunter Comly in the United States in 1945. He reported on two cases and concluded that methemoglobinemia may occur in an infant after ingestion of water high in nitrates, especially if the infant was suffering from a gastrointestinal disturbance (Comly 1945). Fan et al. (1987) have noted since then that microbially poor water (i.e., high in microbes) and high drinking-water nitrate levels often go “hand in hand,” and gastrointestinal illness, as a result of exposure to poor water quality, may play a role in methemoglobinemia.

Nitrate-related drinking-water methemoglobinemia is principally a disease of young children, with bottle-fed or weaned infants < 4 months of age being the most susceptible. This age group is the most susceptible because of a combination of factors (Ayebo et al. 1997), including:

- A higher gastric pH, which allows greater bacterial invasion of the stomach and hence an enhanced conversion of ingested nitrate to nitrite
- A greater fluid intake relative to body weight
- A higher proportion of fetal Hb (which may be more rapidly oxidized to MetHb than adult Hb)
- Lower NADH-dependent MetHb reductase activity (the enzyme that converts MetHb to Hb).

However, although the gastric pH in infants may be higher than that seen in adults, L'hirondel and L’hirondel (2002) have suggested that the general stomach conditions are still not really suitable for the microbial conversion of nitrate to nitrite.

## Exposure Assessment

Methemoglobinemia has several causes, as shown in Table 2, including exposure to nitrite or nitrate through the diet (although high dietary nitrate levels are generally accompanied by high nitrite levels). The principal area of interest in this study, however, was drinking water; therefore, the exposure assessment was based on the concentrations of nitrate in drinking water.

Guidelines and regulatory limits relating to the amount of nitrate in drinking water, of 10 mg/L nitrate–nitrogen (NO_3_–N) and 50 mg/L nitrate [nitrate concentrations are typically expressed either as mg/L NO_3_–N or nitrate (NO_3_); 50 mg/L NO_3_ is equivalent to 11.3 NO_3_–N], were established to prevent infantile methemoglobinemia [U.S. Environmental Protection Agency (EPA) 1977; WHO 1958, 1996], and were based principally on the results of a survey conducted by the American Public Health Association (APHA) and reported by Walton (1951). This survey reported on > 270 cases of methemoglobinemia in infants in the United States (for whom nitrate drinking-water levels were available for 214 cases), although APHA emphasized restricting the data to those cases thought to be definitely associated with nitrate-contaminated water. As noted by Walton (1951), no cases were observed with drinking-water concentrations < 10 mg/L NO_3_–N. High nitrate for the purposes of the exposure assessment has been taken, therefore, to mean anything exceeding the current recommendations.

Natural levels of nitrate in groundwater depend on soil type and geology. In the United States, naturally occurring levels of nitrate are in the range of 4–9 mg/L. Agricultural activities, however, can result in elevated levels (in the region of 100 mg/L; WHO 1996).

High-nitrate drinking water is most often associated with privately owned wells, especially with shallow wells with depths < 15 m in regions with permeable soils (Fan et al. 1987). It is exactly this situation of small community water supplies, in which poorly regulated and unsanitary waters are found, that could induce gastrointestinal symptoms in consumers (Fewtrell et al. 1998). Shearer et al. (1972) note that the factors responsible for elevated nitrate contents in well-water sources include geography, geology, groundwater hydrology, and the addition of nitrates naturally and from surface contamination by nitrogenous fertilizers or by organic waste of human or animal origin. Although water derived from privately owned wells may be the most common source of high-nitrate drinking water, municipal drinking water supplies may also be contaminated. Vitoria Minana et al. (1991) report on nitrate levels in the Valencia region of Spain, where concentrations exceeded the WHO guideline level (50 mg/L) in 95 towns, with 18 municipalities reporting levels > 150 mg/L.

It has been estimated that 15 million families in the United States receive their drinking water from private wells [U.S. General Accounting Office (GAO) 1997]. Assuming an exceedence rate of 13% (based on a survey of 5,500 wells in nine Midwestern states; CDC 1998), an estimated 2 million household supplies would exceed the federal standard of 10 mg/L NO_3_–N. Using current birth rates Knobeloch et al. (2000) estimate that 40,000 infants < 6 months of age are expected to be living in homes with high-nitrate drinking water.

Except for the United States, most literature on nitrate contamination covers small areas and does not allow estimates of the number of people exposed to be calculated.

## Exposure–Response Relationship

Complex co-factor relationships do not currently allow the establishment of a quantitative exposure–response relationship for human exposure to nitrates in food or water and the subsequent development of methemoglobinemia. Two factors make estimates of the number of cases of methemoglobinemia hard to establish: Generally, methemoglobinemia is not a notifiable disease; and definitions of methemoglobinemia (in terms of the required level of MetHb) vary in the literature. Some authors, however, do report incidence rates.

In three counties in Transylvania (Romania), mean incidence rates varied between 117 and 363 of 100,000 live births (for the full 5 years between 1990 and 1994). These rates, reported by Ayebo et al. (1997), were considerably below the previously reported levels of 13,000/100,000 live births, or 13%, which may reflect a decrease in the availability of cheap inorganic fertilizer (hence a decrease in nitrate contamination levels). In 1985, WHO reported that > 1,300 cases of methemoglobinemia (with 21 fatalities) occurred in Hungary over a 5-year period. Indeed, up until the late 1980s methemoglobinemia was a serious problem in Hungary (Hill 1999). Although there are reports of high nitrate concentrations in drinking water (i.e., > 50 mg/L nitrate) from around the world (Hoering and Chapman 2004), these are rarely paralleled by reports of methemoglobinemia. Where illness has been reported, many of the cases predate the early 1990s, and Hanukoglu and Danon (1996) have proposed that the apparent decline in the incidence of methemoglobinemia is suggestive of an infectious etiology.

## Discussion

In addition to the problem of limited data (relating to both the population exposed to nitrate in drinking water and the rate of illness), examination of the literature also revealed a number of factors that would either lead to uncertainty in the disease burden estimate (e.g., avoidance behavior) or cast doubt on the validity of the whole exercise.

### Limited data.

Numerous reports from all over the world describe water supplies (often privately or community-owned wells, rather than municipal supplies) with nitrate concentrations greater than the WHO guideline value of 50 mg/L (Hoering and Chapman 2004). These rarely, however, also include figures on the population supplied by these water sources. Because agricultural and organic waste disposal activities (e.g., through inappropriate sanitation measures) can greatly influence water nitrate concentrations, it is not possible to use geologic data as a possible means to estimate affected population. Thus, it is currently difficult to estimate the population that might be exposed to elevated drinking-water nitrate. Even where the number of people known to have supplies with high nitrate levels can be assessed, this is unlikely to be an accurate estimate of those actually exposed to high-nitrate drinking water. In a number of countries, such as the United States and United Kingdom, health advisories are issued to pregnant women and mothers with formula-fed infants known to be living in high-nitrate areas (Fraser and Chilvers 1981; Schubert et al. 1999). Indeed, Schubert et al. (1999) found that avoidance behavior (i.e., the use of water from another source, such as bottled water or installation of a nitrate removal system) was common, especially in high-risk groups. On the other hand, owners of private wells often boil the water before using it in infant food, an action that, when done excessively, may concentrate nitrate (Ayebo et al. 1997).

A literature review (conducted by searching publication databases and bibliographic lists from collected references) revealed few reported cases of methemoglobinemia linked to water consumption in the last 12 years (Hoering and Chapman 2004). It is possible that because methemoglobinemia is generally not a notifiable disease, there may be under-reporting. It is also possible that there is under-diagnosis, although this is less likely with severe cases, where extensive cyanosis is seen.

### Role of nitrate.

Since the 1940s, when the first cases of methemoglobinemia related to drinking water were reported, there has been the suggestion that gastrointestinal upset, and hence infection, may play a role in the development of the disease (Comly 1945). Comly (1945) suggested that it was advisable to use well water containing no more than 10 or, at the most, 20 mg/L NO_3_–N for infant feeding. This level seemed to be confirmed by the survey conducted by the APHA [cited by Walton (1951)], which suggested that, in instances where drinking-water nitrate had been determined, there were no cases of methemoglobinemia where water concentrations were < 10 mg/L NO_3_–N (~ 45 mg/L nitrate). However, this conclusion would have been influenced by the methodology, which placed an emphasis on cases thought to be linked to nitrate-contaminated water. However, the APHA survey noted that most cases of methemoglobinemia studied were related to NO_3_–N concentrations > 40 mg/L. Additionally, Walton (1951) noted a number of factors that may play a role in the development of infant methemoglobinemia, yet at some point a simple role for nitrate became accepted.

It is becoming increasingly clear, however, that the early authors were correct to be cautious, and now it appears that there is an association between gastrointestinal illness and symptoms of methemoglobinemia in the absence of exogenous nitrate exposure (Bricker et al. 1983; Dagan et al. 1988; Danish 1983; Gebara and Goetting 1994; Kay et al. 1990; Lebby et al. 1993; Smith et al. 1988; Yano et al. 1982). Yano et al. (1982) suggested that diarrhea produces an oxidant stress that increases MetHb production and that acidosis impairs the MetHb reductase systems. Nitric oxide, produced by several tissues in response to infection and inflammation, has also been proposed as a possible mechanism (Gupta et al. 1998; Levine et al. 1998), because nitrite is a product of nitric oxide metabolism. Avery (1999) suggested that exogenous nitrates (e.g., through the consumption of drinking water), rather than causing methemoglobinemia, increase its severity. L’hirondel and L’hirondel (2002) suggested that in cases where methemoglobinemia has been associated with infant formula made with drinking water containing elevated nitrate or carrot soup preparations, it is possible that bacterial growth within the bottle or stored soup and exogenous conversion of nitrate to nitrite is the source of the problem.

## Conclusions

This study did not set out to review the role of nitrates in the causation of methemoglobinemia; however, examination of the literature suggests that a number of authors are starting to question the simple association between nitrate and infant methemoglobinemia, in favor of seeing nitrate as a co-factor in one of several causes of the disease. This factor, coupled with the paucity of data in terms of both population exposure and the level of suspected water-related cases of methemoglobinemia, suggests that attempts to estimate a global burden of disease are currently inappropriate.

## Figures and Tables

**Table 1:** Signs and symptoms of methemoglobinemia.

MetHb concentration (%)	Clinical findings
10–20	Central cyanosis of limbs/trunk
20–45	Central nervous system depression (headache, dizziness, fatigue, lethargy), dyspnea
45–55	Coma, arrhythmias, shock, convulsions
> 60	High risk of mortality

Adapted from Kross et al. (1992).

**Table 2:** Causes of methemoglobinemia.

Designation	Examples
Hereditary	NADH-cytochrome *b*_5_ reductase deficiency, cytochrome *b*_5_ deficiency, M Hb, unstable Hb
Drug/chemical induced	Acetaminophen, amyl nitrite, benzocaine, dapsone, nitroglycerin, nitroprusside, phenazopyridine (pyridium), sulfanilamide, aniline dyes, chlorates, nitrofurans, sulfones
Diet induced	Nitrites, nitrates*^a^*

Adapted from Mansouri and Lurie (1993). M HB is an abnormal type of Hb.

a When followed up, cases have generally been linked to high nitrite levels (e.g., Keating et al. 1973).
